# Timing matters: diagnostic delay and major organ involvement in Behçet’s syndrome

**DOI:** 10.1093/rheumatology/keag528

**Published:** 2026-09-22

**Authors:** Rosaria Talarico, Federica Di Cianni, Antonello Sulis, Diana Marinello, Maria Laura Manca, Marta Mosca

**Affiliations:** Rheumatology Unit, Azienda Ospedaliero-Universitaria Pisana, Department of Clinical and Experimental Medicine, University of Pisa, Pisa, Italy; Rheumatology Unit, Azienda Ospedaliero-Universitaria Pisana, Department of Clinical and Experimental Medicine, University of Pisa, Pisa, Italy; Department of Medical Biotechnologies, University of Siena, Siena, Italy; Rheumatology Unit, Azienda Ospedaliero-Universitaria Pisana, Department of Clinical and Experimental Medicine, University of Pisa, Pisa, Italy; Rheumatology Unit, Azienda Ospedaliero-Universitaria Pisana, Department of Clinical and Experimental Medicine, University of Pisa, Pisa, Italy; Department of Clinical and Experimental Medicine, University of Pisa, Pisa, Italy; Department of Mathematics, University of Pisa, Pisa, Italy; Rheumatology Unit, Azienda Ospedaliero-Universitaria Pisana, Department of Clinical and Experimental Medicine, University of Pisa, Pisa, Italy

**Keywords:** Behçet’s syndrome, diagnostic delay, major organ involvement, disease severity, disease progression, early diagnosis

## Abstract

**Objective:**

To assess the association between diagnostic delay and major organ involvement (MOI) in Behçet’s syndrome (BS), accounting for time at risk and heterogeneity in disease trajectories.

**Methods:**

We conducted a retrospective monocentric cohort study including 173 patients with BS fulfilling ISG and/or ICBD criteria and with at least 5 years of follow-up. Patients were classified according to the presence or absence of MOI (ocular, neurological, gastrointestinal or vascular involvement). Diagnostic delay was defined as the time from symptom onset to diagnosis. Logistic regression and sensitivity models assessed the association between diagnostic delay and cumulative MOI, accounting for age at onset, sex and disease duration. Time-to-event analyses were performed among patients without MOI at disease onset to evaluate incident MOI during follow-up.

**Results:**

Among 173 patients, 101 (58.4%) had at least one cumulative MOI. Timing of first MOI was available for 100 patients: 67 had MOI already present at disease onset, whereas 33 developed incident MOI during follow-up. Diagnostic delay was longer in patients with cumulative MOI than in those without MOI. In the initial logistic regression model adjusted for age at onset and sex, diagnostic delay was associated with cumulative MOI; however, this association was attenuated after accounting for disease duration. Time-to-event analysis among patients without MOI at disease onset did not show a statistically significant association between diagnostic delay and incident MOI.

**Conclusions:**

Diagnostic delay was associated with cumulative MOI in BS; however, this association was attenuated after accounting for disease duration and time at risk. The findings support the existence of heterogeneous disease trajectories, including early severe disease, incident MOI during follow-up and absence of MOI over the observed period. These results should be interpreted as hypothesis-generating and require confirmation in prospective longitudinal studies.

*Rheumatology* key messages

- Accounting for time at risk is essential when interpreting diagnostic delay and organ involvement associations.
- Behçet’s syndrome follows heterogeneous trajectories, including early major organ involvement, later involvement or persistently non-major disease.
- Early recognition remains important, although prospective studies must determine whether reducing diagnostic delay modifies outcomes.

## Introduction

Behçet’s syndrome (BS) is a complex, multisystem inflammatory disorder characterized by marked clinical heterogeneity, ranging from mucocutaneous-limited manifestations to severe organ-threatening involvement affecting the eyes, CNS, gastrointestinal tract and vascular system [1–3]. Understanding the determinants of disease severity remains a major challenge in clinical practice [4–6]. In the last years, two main conceptual frameworks have been proposed to explain this heterogeneity. On one hand, phenotype-based approaches have identified distinct clusters of clinical manifestations, suggesting that BS may consist of different disease phenotypes rather than a single uniform entity [7]. In this context, previous analyses from our group using multivariate techniques highlighted the existence of distinct clinical patterns of organ involvement, supporting the concept of phenotype-driven disease expression [8]. However, the reproducibility and clinical robustness of phenotype clustering remain debated. A recent systematic review highlighted substantial variability in phenotype definitions across studies, even within similar populations, largely driven by methodological differences, thus questioning the stability and generalizability of phenotype-based classifications [9]. On the other hand, large multicentre studies, including data from international registries, have focused on identifying predictors of progression from mucocutaneous-limited disease to major organ involvement (MOI), reinforcing the concept of a temporal disease trajectory and enabling risk stratification of patients at higher risk of severe outcomes [10]. Taken together, these approaches have significantly advanced our understanding of BS heterogeneity; however, they largely consider disease severity as an intrinsic feature of the disease, either determined by underlying biological heterogeneity or by predefined progression pathways. These observations suggest that neither phenotype-based nor progression-based models fully capture the determinants of disease severity in BS. Moreover, diagnostic delay is a well-recognized issue in BS, due to its heterogeneous presentation and the absence of specific diagnostic biomarkers. Prolonged intervals between symptom onset and diagnosis may reflect delayed disease recognition, heterogeneous referral pathways or delayed access to specialist assessment and may be associated with differences in disease monitoring and management [1, 11–17]. Whether diagnostic delay is associated with MOI, independently of intrinsic disease characteristics and time at risk, remains unclear.

In this study, we aimed to investigate the association between diagnostic delay and MOI in a monocentric cohort of patients with BS, while accounting for time at risk and heterogeneity in disease trajectories.

## Methods

### Study design and setting

This was a retrospective, monocentric cohort study conducted at a tertiary referral centre with dedicated expertise in BS. Patients were included if they fulfilled the International Study Group (ISG) and/or the International Criteria for Behçet’s Disease (ICBD) classification criteria and had a minimum follow-up of 5 years. A total of 173 patients with a confirmed diagnosis of BS were included. Clinical and demographic data were retrospectively collected from medical records and the local database.

### Ethical approval

The study was approved by the Tuscany Regional Ethics Committee (CERT-P), protocol code n. 122/2024, approved on 09/07/2024. Written informed consent was obtained from all participants.

### Definition of MOI

Patients were classified according to the presence or absence of MOI. MOI was defined as a history of ocular, neurological, gastrointestinal or vascular involvement, based on clinical evaluation and, when appropriate, imaging or specialistic assessment. Patients without any history of MOI during follow-up were classified as patients without MOI during the observed follow-up.

### Data collection

The following variables were collected: sex, age at disease onset, age at diagnosis, disease duration, follow-up duration, diagnostic delay and cumulative clinical manifestations. Diagnostic delay was defined as the time interval between disease onset and diagnosis. Disease onset was defined as the first patient-reported symptom subsequently attributed to BS and recorded in the medical chart after specialist assessment. When multiple symptoms were reported, the earliest symptom considered compatible with BS was used. Clinical manifestations were assessed cumulatively over the disease course. For patients with MOI, the timing of first organ involvement and the occurrence of subsequent MOI were also recorded to explore temporal patterns of disease evolution. Detailed data on treatment exposure before diagnosis, timing of treatment initiation and treatment intensity over follow-up were not consistently available in the retrospective dataset and were therefore not included in the main analyses.

### Statistical analysis

Continuous variables were reported as mean ± S.D. or median, as appropriate, while categorical variables were expressed as frequencies and percentages. Comparisons between patients with and without MOI were performed using the *χ*^2^ test or Fisher’s exact test for categorical variables, and Student’s *t*-test or Mann–Whitney *U*-test for continuous variables, according to data distribution. To assess the association between diagnostic delay and cumulative MOI, logistic regression analysis was performed with MOI as the dependent variable and diagnostic delay as the main independent variable, adjusting for age at disease onset and sex. Results were reported as odds ratios (ORs) with 95% CIs. Additional sensitivity models including disease duration and/or follow-up duration were performed to evaluate the potential impact of time-at-risk bias.

To address potential time-at-risk bias, additional time-to-event analyses were performed. Patients with MOI already present at disease onset were analyzed separately from those developing incident MOI during follow-up. Among patients without MOI at disease onset, Cox proportional hazards regression was used to assess the association between diagnostic delay and time to first incident MOI. Patients who did not develop MOI were censored at last follow-up. In addition, an exploratory landmark analysis was performed from the time of diagnosis among patients without MOI at diagnosis, evaluating post-diagnosis incident MOI. Kaplan–Meier curves were generated to visually describe cumulative incidence of incident MOI.

A secondary descriptive longitudinal analysis was conducted to explore the timing of first MOI and progression from single- to multi-organ involvement. Exploratory organ-specific analyses were also performed for ocular, neurological, vascular and gastrointestinal involvement. A two-sided *P*-value <0.05 was considered statistically significant.

## Results

### Patient characteristics and disease trajectories

A total of 173 patients with BS were included, of whom 101 (58.4%) had at least one cumulative MOI and 72 (41.6%) had no MOI during the observed follow-up. Timing of first MOI was available for 100 of 101 patients with cumulative MOI. Among these, 67 patients had MOI already present at disease onset, whereas 33 developed incident MOI after disease onset. One patient with cumulative MOI had unavailable timing of first MOI and was therefore included in the cumulative MOI analyses but excluded from trajectory-based and time-to-event analyses.

Patients were therefore classified into three trajectory groups: MOI at disease onset, incident MOI after disease onset and no MOI during the observed follow-up. The demographic and clinical characteristics of these groups are shown in Table 1. Patients developing incident MOI after disease onset had the longest diagnostic delay, whereas patients without MOI had shorter disease duration and follow-up.

**Table 1:** Demographic and clinical characteristics according to disease trajectory.

Variable	MOI at disease onset (*n* = 67)	Incident MOI after disease onset (*n* = 33)	No MOI during observed follow-up (*n* = 72)
Male sex, *n* (%)	23 (34.3)	13 (39.4)	18 (25.0)
Female sex, *n* (%)	44 (65.7)	20 (60.6)	54 (75.0)
Age at disease onset, years, mean ± S.D.	30.52 ± 11.66	27.24 ± 10.36	32.22 ± 11.97
Age at diagnosis, years, mean ± S.D.	36.64 ± 10.91	37.27 ± 10.80	36.06 ± 11.88
Diagnostic delay, years, mean ± S.D.	6.12 ± 8.46	10.03 ± 9.10	3.83 ± 4.97
Diagnostic delay, years, median (IQR)	3.00 [0.00–7.50]	6.00 [3.00–17.00]	1.50 [0.00–6.00]
Disease duration, years, mean ± S.D.	21.12 ± 9.71	25.70 ± 11.10	14.92 ± 8.62
Follow-up duration, years, mean ± S.D.	15.00 ± 7.48	15.67 ± 8.99	11.08 ± 6.55
Time to first MOI, years, median (IQR)	0.00 [0.00–0.00]	11.00 [7.00–18.00]	–

Abbreviations: IQR: interquartile range; MOI: major organ involvement.

### Diagnostic delay and MOI

Diagnostic delay was significantly longer in patients with cumulative MOI than in those without MOI during the observed follow-up (7.34 ± 8.68 vs 3.83 ± 5.18 years; *P* = 0.0011). A similar difference was confirmed using non-parametric analysis (median 4.0 vs 1.5 years; Mann–Whitney *P* = 0.0108).

In the initial logistic regression model adjusted for age at disease onset and sex, diagnostic delay was associated with cumulative MOI (OR 1.07 per year, 95% CI 1.02–1.13; *P* = 0.011), whereas age at onset and sex were not significantly associated with MOI. However, when disease duration was added to the model to account for differences in time at risk, the association between diagnostic delay and cumulative MOI was attenuated and was no longer statistically significant, whereas disease duration remained associated with cumulative MOI. These findings suggest that the association observed in the cumulative analysis was at least partly influenced by longer disease duration and greater opportunity to detect organ involvement over time. The results of the regression and time-to-event analyses are summarized in Table 2.

**Table 2:** Regression and time-to-event analyses assessing the association between diagnostic delay and major organ involvement.

Analysis	Population	Outcome	Diagnostic delay estimate	95% CI	*P*-value	Interpretation
Initial logistic regression model adjusted for age at onset and sex	All patients, *n* = 173	Cumulative MOI	OR 1.07	1.02–1.13	0.011	Diagnostic delay was associated with cumulative MOI in the initial model
Logistic regression additionally adjusted for disease duration	All patients, *n* = 173	Cumulative MOI	OR 0.99	0.93–1.06	0.801	Association attenuated after accounting for disease duration
Cox regression from disease onset among patients without MOI at onset	*n* = 106; events = 33	First incident MOI	HR 1.03	0.99–1.07	0.197	No statistically significant association with incident MOI
Exploratory landmark Cox regression from diagnosis among patients without MOI at diagnosis	*n* = 93; events = 20	Post-diagnosis incident MOI	HR 1.09	1.01–1.16	0.020	Exploratory association; interpreted cautiously due to limited events

Diagnostic delay was analyzed as a continuous variable. The landmark Cox analysis was considered exploratory because of the limited number of post-diagnosis incident MOI events.

Abbreviations: MOI: major organ involvement; HR: hazard ratio; OR: odds ratio.

### Time-to-event analysis of incident MOI

To further address time-at-risk bias, we performed time-to-event analyses restricted to patients without MOI at disease onset. In this subgroup, Cox regression analysis from disease onset did not show a statistically significant association between diagnostic delay and time to first incident MOI (HR 1.03, 95% CI 0.99–1.07; *P* = 0.197). Kaplan–Meier curves were generated to describe the cumulative incidence of incident MOI over time according to diagnostic delay group (Fig. 1).

**Figure 1:**
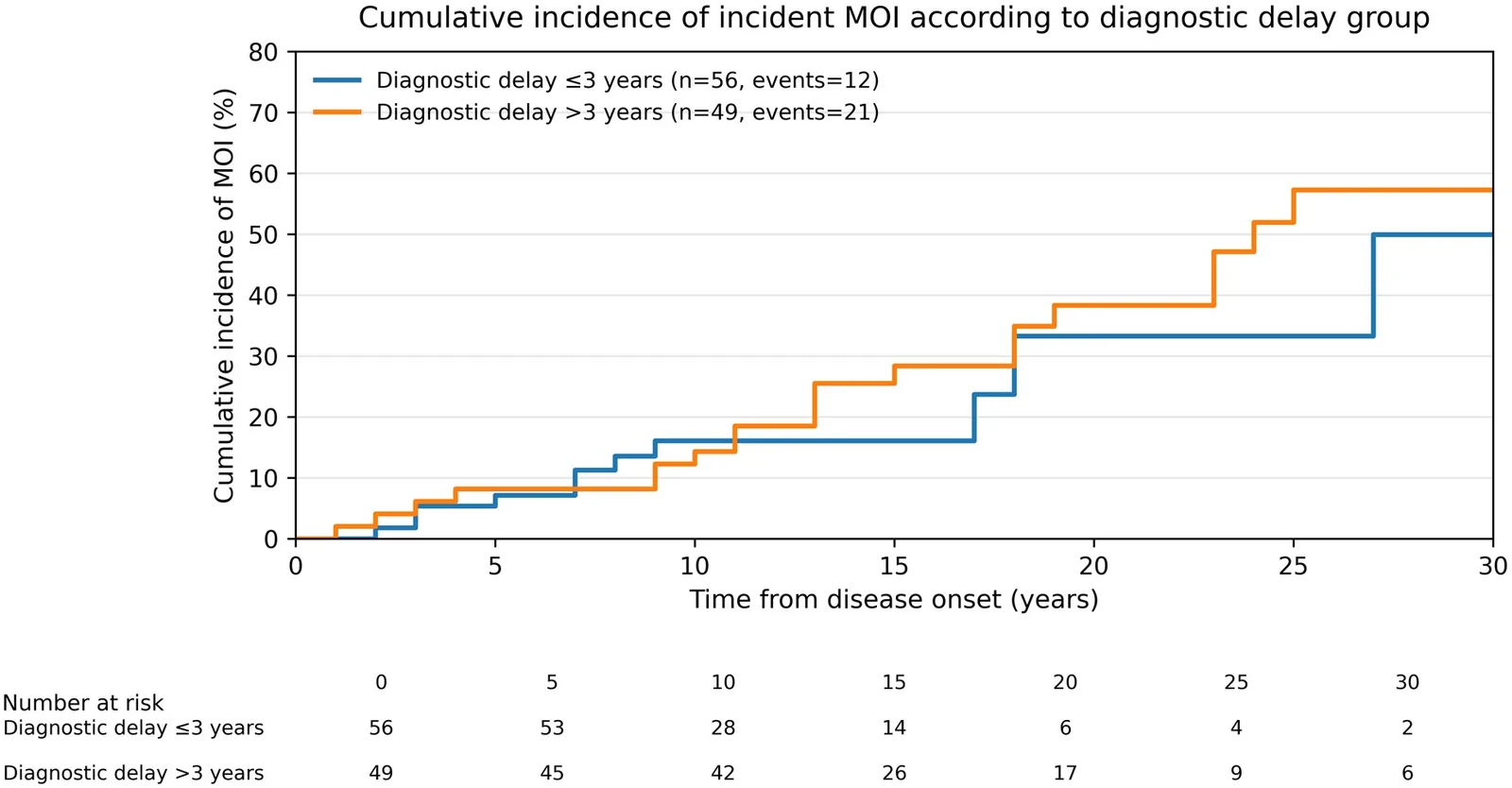
Cumulative incidence of incident MOI according to diagnostic delay group. Analysis restricted to patients without MOI at disease onset. Patients without incident MOI were censored at last available follow-up. Diagnostic delay was stratified according to the cohort median (≤3 vs >3 years)

In an exploratory landmark analysis restricted to patients without MOI at diagnosis, longer diagnostic delay was associated with post-diagnosis incident MOI (HR 1.09, 95% CI 1.01–1.16; *P* = 0.020). However, given the limited number of events, this finding should be interpreted cautiously and considered hypothesis-generating (Supplementary Fig. S1).

### Timing and type of first MOI

Among the 100 patients with available timing of first MOI, 67 had MOI already present at disease onset, whereas 33 developed incident MOI during follow-up. Among patients who developed incident MOI after disease onset, timing was heterogeneous, with some cases occurring within the first 5 years and others emerging >10 years after disease onset. Overall, ocular involvement was the most frequent first MOI, followed by neurological and vascular involvement. Exploratory organ-specific descriptive analyses were performed to account for the heterogeneity of the composite MOI end point. Ocular involvement was the most frequent organ-specific MOI and was commonly present at disease onset, although incident ocular involvement was also observed during follow-up. Neurological, vascular and gastrointestinal involvement showed more heterogeneous timing patterns, with both onset and incident cases. Given the limited number of events for some organ-specific outcomes, these analyses were considered descriptive and hypothesis-generating. The results are reported in Supplementary Table S1.

### Progression to multi-organ involvement

Among patients with MOI, 26 developed involvement of a second major organ, and six progressed to a third MOI. In patients with sequential progression, the median time from first to second MOI was 4.0 years. Ocular and vascular manifestations represented the most frequent transition pattern, suggesting recurrent patterns of disease evolution.

### Evolution of clinical manifestations over time

Longitudinal analysis showed that several severe manifestations increased over time in the MOI group. Ocular involvement rose from 49.5% at onset to 76.2% cumulatively, parenchymal CNS involvement from 5.9% to 23.8%, superficial thrombophlebitis from 7.9% to 18.8% and deep vein thrombosis from 5.0% to 12.9%. Endoscopy/imaging-confirmed gastrointestinal involvement also emerged during follow-up, reaching 10.9% cumulatively. By contrast, differences in mucocutaneous manifestations between the groups tended to attenuate over time.

### Patients without MOI during observed follow-up

Patients classified as without MOI did not develop neurological, vascular, gastrointestinal or clinically significant ocular involvement during the available follow-up. However, given their shorter disease duration and follow-up compared with patients with MOI, this finding should not be interpreted as definitive evidence of a permanently benign trajectory.

## Discussion

The present study explored the association between diagnostic delay and MOI in a monocentric retrospective cohort of patients with BS. In the initial cumulative analysis, diagnostic delay was associated with MOI. However, this association was attenuated after accounting for disease duration, indicating that differences in time at risk substantially contributed to the observed relationship. In addition, time-to-event analyses restricted to patients without MOI at disease onset did not confirm a statistically significant association between diagnostic delay and incident MOI. These findings suggest that the relationship between diagnostic delay and MOI is more complex than initially hypothesized and should be interpreted in the context of heterogeneous disease trajectories, differences in follow-up duration and potential residual confounding. Traditionally, BS has been interpreted through two main conceptual frameworks: phenotype-based models and progression-based models. Phenotype-based approaches, including previous analyses from our group, have identified distinct clusters of clinical manifestations, supporting the concept of phenotype-driven disease expression [8]. However, recent evidence has highlighted substantial variability in phenotype definitions across studies, raising concerns regarding their reproducibility and generalizability [9]. On the other hand, large multicentre studies have focused on identifying predictors of progression to MOI, reinforcing the concept of a temporal disease trajectory and enabling risk stratification [10–11, 13, 15]. While these models have significantly advanced the understanding of BS heterogeneity, they largely consider disease severity as an intrinsic feature of the disease. These findings should also be interpreted within the established natural history of BS. The occurrence of MOI is not uniform over time, and different organ manifestations may follow distinct temporal patterns [10, 11, 15, 18]. Ocular and vascular involvement are often recognized as major determinants of severity, particularly during earlier disease phases and in patients with high-risk clinical features [11, 13, 15]. Conversely, patients who remain free from MOI during the early disease course may have a lower subsequent probability of developing major involvement, although late incident MOI can still occur [18]. Therefore, diagnostic delay should not be viewed as a uniform driver of MOI, but rather as a factor potentially interacting with intrinsic disease phenotype, referral pathways, monitoring, treatment exposure and time at risk. Our findings support a trajectory-based interpretation of MOI in BS. The distinction between MOI already presents at disease onset and incident MOI developing during follow-up is clinically relevant. Patients with MOI at onset may represent an early severe trajectory, likely driven by intrinsic disease phenotype and biological susceptibility. Conversely, patients developing MOI during follow-up may represent a progressive trajectory in which time-dependent factors, including diagnostic delay, referral pathways, disease monitoring and treatment exposure, may contribute to disease evolution. However, because of the retrospective design and the lack of detailed treatment timing data, this interpretation should be considered hypothesis-generating. The temporal dimension of disease evolution observed in our cohort suggests that BS follows heterogeneous trajectories. While a substantial proportion of patients already present MOI at disease onset, a relevant subset develops organ involvement during follow-up, with progression to multi-organ disease occurring in approximately one quarter of cases. In contrast, patients without MOI did not develop MOI during the observed follow-up, although they had shorter disease duration and follow-up compared with patients with MOI. Therefore, absence of MOI in this group should not be interpreted as evidence of a permanently non-severe trajectory. Overall, these findings support the existence of heterogeneous disease trajectories, including MOI at disease onset, incident MOI during follow-up and absence of MOI during the observed period. Previous studies from our group have shown that the absence of MOI in the early years of BS does not necessarily predict a favourable long-term outcome, as a proportion of patients may still develop *de novo* major involvement during follow-up [18]. These findings highlighted the dynamic nature of disease evolution. The present study builds on this concept by suggesting that diagnostic delay may be considered as one potential component of a broader disease and healthcare pathway associated with cumulative MOI. Taken together, these observations suggest that cumulative MOI in BS may reflect the interplay between intrinsic disease phenotype, time at risk, referral pathways and other unmeasured factors, including treatment exposure. Therefore, MOI should not be interpreted as a direct consequence of diagnostic delay. Rather, diagnostic delay may represent one component of a broader and more complex disease and healthcare pathway. From a clinical perspective, these findings still support the importance of early recognition and timely referral of patients with suspected BS, particularly for comprehensive assessment of possible organ involvement at or near disease onset [19–21]. However, our data do not demonstrate that reducing diagnostic delay prevents MOI or modifies disease trajectories. The differing results between the Cox analysis from disease onset and the exploratory landmark analysis from diagnosis may reflect differences in the time origin, selected at-risk populations and number of incident events considered in each model. In particular, the landmark analysis focused only on post-diagnosis events among patients without MOI at diagnosis and should therefore be interpreted as exploratory and hypothesis-generating. This hypothesis requires prospective longitudinal studies incorporating treatment timing, referral pathways and standardized assessment of incident organ involvement. This study has some limitations. Its retrospective and monocentric design may limit generalizability, and residual confounding factors cannot be excluded. In addition, the tertiary referral setting may have introduced referral bias, with potential enrichment for patients with more severe, atypical or diagnostically complex disease. Diagnostic delay may be subject to recall bias, particularly in patients with long disease duration, as the timing of first symptoms was retrospectively reconstructed from clinical records and patient history. Some patients may also have been evaluated or managed by other specialists before referral to our centre, including dermatologists, ophthalmologists, neurologists, gastroenterologists or vascular physicians, which may have influenced both diagnostic delay and the timing of MOI recognition. In particular, although the temporal relationship between diagnostic delay and MOI is biologically plausible, causality cannot be definitively established. Moreover, detailed data on treatment exposure before diagnosis, timing of treatment initiation and treatment intensity over follow-up were not consistently available. Therefore, diagnostic delay should not be interpreted as synonymous with treatment delay or as a direct proxy for untreated disease. Treatment exposure before formal BS diagnosis may have varied considerably among patients, as some may have received symptomatic or specialist-directed treatment, whereas others may have remained untreated despite symptoms. Consequently, we could not assess whether the association between diagnostic delay and MOI was mediated or modified by treatment exposure. BS patients with MOI also had significantly longer disease duration and follow-up, which may have increased the opportunity to capture cumulative organ manifestations over time. Therefore, the observed association between diagnostic delay and MOI should be interpreted with caution, as it may be partially influenced by time-at-risk. Future studies using time-to-event analyses are warranted to better clarify this relationship. Finally, although patients with MOI at disease onset and those developing incident MOI during follow-up were analyzed separately in the revised analyses, the retrospective nature of the study and the limited number of incident events may still have influenced the precision and interpretation of trajectory-specific findings. In conclusion, diagnostic delay was associated with cumulative MOI in BS in initial analyses, but this association was attenuated after accounting for disease duration and time at risk. Our findings support the existence of heterogeneous disease trajectories rather than a uniform relationship between diagnostic delay and disease severity. Early recognition and referral remain clinically important, but prospective longitudinal studies incorporating treatment timing and referral pathways are needed to determine whether reducing diagnostic delay may modify the subsequent occurrence of MOI.

## Supplementary Material

keag528_Supplementary_Data

## Data Availability

Data are available upon reasonable request from the corresponding author.

## Notes

*Disclosure statement:* The authors have no conflict of interest to declare.

## Notes

*Patient and public involvement*: Patients and/or the public were not involved in the design, conduct, reporting or dissemination plans of this research.
