# Supplementary material for: Timing matters: diagnostic delay and major organ involvement in Behçet’s syndrome
Source: Rheumatology (Oxford). 2026 Sep 22;65(10):keag528. doi: 10.1093/rheumatology/keag528 (PMC13637606; doi:10.1093/rheumatology/keag528)
Supplement: Supplementary file 1 [file keag528_supplementary_data.docx]

| **Organ-specific MOI** | **Total events, n** | **Present at disease onset, n** | **Incident after disease onset, n** | **Median time to incident involvement, years** |
| --- | --- | --- | --- | --- |
| Ocular involvement | 75 | 51 | 24 | 8.5 |
| Neurological involvement | 22 | 5 | 17 | 11.0 |
| Vascular involvement | 25 | 12 | 13 | 7.0 |
| Gastrointestinal involvement | 10 | 3 | 7 | 15.0 |

**Supplementary Table S1. Organ-specific timing of major organ involvement.** MOI: major organ involvement. Analyses were descriptive and exploratory because of the limited number of events for some organ-specific outcomes.

**Supplementary Figure S1**. Post-diagnosis cumulative incidence of incident major organ involvement (MOI) according to diagnostic delay group. Landmark analysis restricted to patients without MOI at diagnosis. Patients without post-diagnosis incident MOI were censored at last available follow-up after diagnosis. Diagnostic delay was stratified according to the cohort median (≤3 versus >3 years).

Supplementary Figure S1 alt text: *Post-diagnosis cumulative incidence curves of incident major organ involvement (MOI) in patients with Behçet’s syndrome, stratified by diagnostic delay ≤3 years versus >3 years. After diagnosis, the two groups show broadly similar long-term cumulative incidence patterns, with some early separation between the curves.*
